# Conductivity of Filled Diblock Copolymer Systems: Identifying the Main Influencing Factors

**DOI:** 10.3390/polym17111502

**Published:** 2025-05-28

**Authors:** A. I. Chervanyov

**Affiliations:** Institute of Theoretical Physics, University of Münster, 48149 Münster, Germany; chervany@uni-muenster.de

**Keywords:** diblock copolymers, fillers, conduction, 83.80.Uv, 82.35.Np

## Abstract

By developing and making use of the multi-scale theoretical approach, we identify the main factors that affect the conductivity of a composite composed of a diblock copolymer (DBC) system and conductive particles. This approach relies on the consistent phase-field model of DBC, Monte-Carlo simulations of the filler localization in DBC, and the resistor network model that mimics the conductive filler network formed in DBC. Based on the described approach, we thoroughly explore the relation among the morphological state of the microphase-separated DBC, localization of fillers in DBC, and the electrical response of the composite. Good agreement with experimental results confirms the accuracy of our theoretical predictions regarding the localization of fillers in the DBC microphases. The main factors affecting the composite conductivity are found to be: (i) affinities of fillers for copolymer blocks; (ii) degree of the segregation of a host DBC system, driven by external stimuli; (iii) geometry of the microphases formed in the microphase-separated DBC; and (iv) interactions between fillers. The conductor-insulator transition in the filler network is found to be caused by the order-disorder transition in the symmetric DBC. The order-order transition between the ordered lamellae and cylindrical microphases of asymmetric DBC causes a spike in the composite conductivity.

## 1. Introduction

The rising trend of replacing conventional solid materials used in electronic devices with soft counterparts has led to the establishment of the new field of soft electronics. Soft electronic devices, primarily based on polymeric materials, exhibit remarkable sustainability under complex working conditions involving deformations, stretching, or folding. The beneficial properties of these soft materials can be further enhanced by adding solid fillers. Fillers are known to significantly improve the thermodynamic [1,2,3], tensile [4], and rheological [5] properties of many polymer-based composites. This makes filled polymer systems promising candidates for use in a variety of practical electromechanical applications, such as micro-electromechanical systems [6,7], sensors and actuators [8,9,10,11,12,13,14], and batteries [15,16].

In the mentioned electrical applications, the primary factor affecting the conductivity of polymer composites filled with conductive fillers is the formation of the filler network within these composites [4,17]. The possibility to indirectly alter the structure of a filler network by manipulating the soft matrix containing this network provides a variety of efficient routes to tailoring the electrical properties of filled polymer composites. Depending on the nature of the host polymer matrix, one can apply different external stimuli (e.g., temperature, stress) [5,18,19,20] or manipulate the intrinsic properties of composite components (e.g., surface chemistry of fillers [21] or polymer-filler interactions [4,17,22,23]) to gain control over the electrical response of this composite. The controllable driving of this electrical response, in turn, can be used to induce the conducting or insulating state of composites, which can be employed, in particular, in soft mechanical-electrical sensors.

In a recent series of papers [24,25,26,27] featured in the present work, we proposed a novel approach for driving the electrical response of filled polymer-particle composites by using diblock copolymers (DBC) as an insulating host polymer system containing highly conductive fillers. Unlike other multi-component polymer systems (e.g., polymer blends), DBC can form geometrically well-organized, nano-sized morphological features (e.g., lamellae, cylinders, spheres, and gyroids), which are often used in various applications [28,29]. The unique advantage of the DBC system lies in its ability to alter its morphology in response to changing external conditions [30,31,32]. One important example of this effect is the alteration of DBC morphology in response to temperature changes. Depending on the composition *f* of DBC, reducing temperature below its order-disorder transition (ODT) value results in the formation of either lamellar or cylindrical morphology. For asymmetric DBC, further decreasing temperature below ODT results in the order-order transition between the cylindrical and lamellar morphologies. When DBC system is filled with particles, the localization of these fillers is known [2,33,34,35,36,37,38] to drastically depend on the DBC morphology. The described transitions between the DBC morphologies can be, therefore, used to tailor the selective localization of fillers in the formed morphological structures [2,36,37,38].

One of the conventional methods for controlling the conductivity of composites by altering the localization of fillers is the application of mechanical deformations, such as external stress [5,18]. This approach is commonly used, for example, in strain sensors [19], but it presents additional technical challenges, such as the need for precise control over deformations or ensuring the stability of the conductive network under high-strain conditions. The present work explores an alternative route to achieving the desired localization of fillers and, thereby, the conductivity of composites. As shown in the present work, the key factor that affects the conductivity of the described filled DBC system is the localization of fillers in the microphase domains of this system and interfaces between them. As demonstrated in [24,26], this localization is largely dependent on the morphology of the host DBC system. This feature opens up a way to control composite conductivity in general, and to optimize conductive thresholds in particular, by tuning the DBC molecular weight or DBC block ratios that determine the DBC morphology at a given temperature. This novel approach can be especially useful in the preparation of composites with predetermined non-uniform filler localization for use in electrical sensors.

Although filled DBC systems have been studied theoretically by several methods in the previous work described below, the localization of fillers is still not completely understood, even for the simple lamellar morphology of DBC. Moreover, to the best of the author’s knowledge, the theoretical findings reported in previous work have been never compared against experimentally observed distribution of fillers in the DBC microphases. This gap between previous theories and experiment hinders the evaluation of the adequacy of the developed theoretical concepts. In contrast, in the present work we perform the comparison with experimental results described in Section 3.2.1 to verify our theoretical predictions of filler localization in the lamellar DBC morphology. This comparison demonstrates the high accuracy of the simulations performed.

Significant progress in the understanding of the filler localization has been made by the Self Consistent Field-Density Functional Theory (SCFT-DFT) [38,39]. In its original form, this theory relies on the continuum field description of the filler particles, similar to the mean-field description of the polymer species embedded in SCFT for pure (unfilled) DBC. Although this pioneering approach provides a tool for describing the coarse-grained macro-scale picture of the distribution of species in filled DBC, it lacks microscopic details critical for the current analysis. In particular, this approach does not make it possible to describe the excluded volume (steric) interactions between fillers and polymers. In the present work we show that its these interactions that underlie the preferential localization of fillers at the interfaces between the DBC microphases observed in experiment [40], and they therefore cannot be neglected. The steric interactions between fillers are described in SCFT-DFT by directly adding the local Carnahan-Starling term to the free energy density of the composite. Furthermore, the enthalpic interaction between fillers and polymers is described by the simplistic Flory-Huggins approach with constant χ-parameters that do not depend on the filler size and local DBC composition. As we show in the current work, the true surface interactions between fillers and polymers, when rigorously taken into account, prove to be the key to understanding the selective localization of fillers in the DBC microphases. This effect essentially depends on the filler radius, as well as the *local* composition of DBC in the vicinity of the fillers. These dependencies, critical for understanding the filler localization, are not captured by the mean field description of the polymer-filler interactions adopted in SCFT-DFT.

The deficiencies of the described SCFT-DFT theory are partially remedied by a family of hybrid methods [41,42,43] that model fillers as true three-dimensional spherical particles. The theoretical development in [43] relies on the Brownian dynamic simulation of fillers immersed in DBC, described by the standard SCFT approach. The effect of the DBC morphology on the dynamics of fillers is reduced to the force fields derived from the simulated fluctuating chemical potential of the DBC species, which are updated at each time step of the simulation. A more recent version [42,44] of the hybrid method relies on a similar simulation scheme for filler particles, while DBC are described by the Ohta-Kawasaki model [45]. The interactions between DBC and fillers in this approach are described at the thermodynamic level, by the additional contribution to the system’s free energy caused by the presence of fillers. This contribution is postulated by using the simplistic artificial “tagged function” that couples the filler localization to the DBC morphological structure. The treatment of polymer-filler interactions in the described hybrid approaches, therefore, still does not account for the excluded volume interactions. Recall that accounting for these interactions is critical for understanding the interfacial filler localization observed in experiments [40], as mentioned in the preceding paragraph. In addition, the significant conceptual improvements provided by the SCFT-based hybrid method in [43] come at the cost of high computational demands, which can limit its accuracy [41]. The alternative method developed in [41] is based on the concept of “cavity functions” representing fillers. These functions are considered in the ideal limit, so the predictions of filler localization can be considered reasonably accurate only for filler volume fractions of less than $\phi_{c}∼$1%, according to the authors of [41]. This method is therefore also inapplicable to the conductivity calculations performed in the present work, which show that the filler electrical percolation threshold lies higher than $\phi_{c}$.

The determination of the preferential localization of fillers constitutes a key part of the multiscale approach developed in the current work. This approach relies on the continuum phase-field model of the microphase-separated DBC, coupled with Monte Carlo (MC) simulations of the localization of fillers in this system. The method used to describe the filler system is well-suited for calculating the composite conductivity. Specifically, the localization of fillers, simulated by MC, is used to construct the random resistor network that mimics the actual filler network formed in a given DBC morphology.

From the perspective of its performance in the conductivity calculations, the proposed approach offers several conceptual advantages over the SCFT-DFT and hybrid approaches described above. In particular, our approach avoids the use of the continuum field representation for the filler system employed in SCFT-DFT [38]. Furthermore, the developed phase-field model extends beyond the Flory–Huggins approximation, which is restricted to second-order terms in the densities of polymer species, as adopted in SCFT-DFT. Moreover, we do not employ any artificial models for hard fillers, e.g.,“tagged function” representation of fillers adopted in [44]. Instead, we consider actual finite-size filler particles that experience the excluded volume and surface adsorption interactions with DBC. The surface adsorption interactions are quantified by the adhesion energy, which can be directly obtained from experimental data, thereby establishing a direct link between the developed theory and experiment. As we demonstrate in what follows, the described excluded volume and adsorption interactions are responsible for the preferential localization of fillers at the interfaces and within the selective DBC microphases, respectively. The relative significance of these interactions is quantified by the filler immersion energy, rigorously calculated by the developed phase-field model of filled DBC. The obtained filler immersion energy proves to be the key factor determining the localization of fillers. This localization, in turn, is shown to determine the conductivity of a filled DBC system, depending on the prevailing polymer-filler interaction and the morphology adopted by DBC. Finally, it is important to note that the proposed approach, which is based on Monte Carlo simulations of the filler system, does not entail the high computational cost associated with the Brownian dynamics simulations used in the hybrid methods. This feature is critical for the performed conductivity calculations, which rely on repeated simulations of a relatively large filler network having a density above the percolation threshold.

The paper is organized as follows: In Section 2, the multiscale approach developed and used to calculate the conductivity of a filled DBC system is described. Section 2.2 presents the phase field model of the DBC system in an external field. Section 2.3 uses this model to derive the immersion energy of a filler immersed in the microphase-separated DBC system. Section 2.4 discusses the Monte Carlo simulations applied to predict the preferential localization of fillers in the microphases of the DBC. Section 2.5 calculates the conductivity of the composite using the developed random resistor network model. The results obtained in Section 2 are analyzed and discussed in Section 3. Section 3.2 describes the simulation results of the localization of fillers in DBC, while Section 3.3 presents the results of the conductivity calculations for the filled DBC system. Subsequent subsections of these sections separately examine the effects of the difference in affinities of fillers for dissimilar copolymer blocks, interactions between fillers, changes in composite temperature, and alterations in the microphase morphology of DBC. Section 4 provides the conclusions and outlook.

## 2. Multiscale Model of the Conductivity of Filled DBC System

### 2.1. Structure of the Model

To describe the conductivity of an insulating DBC system filled with conductive fillers, we have developed a multi-scale approach that encompasses the four stages described in the following subsections. The first stage involves developing a phase-field model of a filled DBC system in an external field. Technically, this stage requires extensive numerical work to solve the phase-field equations that describe the morphology of the DBC system. In the second stage, the immersion energy of filler particles is calculated to provide input for predicting the probability of a given localization of fillers for each DBC morphology. Note that this stage addresses the smallest microscopic scale of the system, as it focuses on the true microscopic interactions between fillers and polymers. The output from the first two stages is then fed into the third stage: lattice Monte Carlo simulations predicting the most probable localization of fillers in the DBC system. The final, fourth stage of the approach involves calculating the conductivity of the composite, which consists of an insulating DBC system and conductive fillers. The main input for this calculation is the localization of filler particles in the DBC system for each DBC microphase morphology obtained in the preceding stages. The conductivity calculation is performed by combining the bond percolation model and the resistor network model. In the following subsections, we outline the detailed workflow of the approach developed in [25,26,27].

### 2.2. Unfilled DBC System in an External Field: Phase Field Model

We start by considering a system of identical linear DBC having polymerization degree *N*. Each copolymer is comprised of two chemically uniform blocks containing fN of the *A*-monomers and (1−f)N of the *B*-monomers, *f* being the fraction of the *A*-monomers in each copolymer chain. We restrict the consideration to dense DBC systems that are known [46] to be incompressible to a good approximation. Owing to the incompressibility constraint, the considered DBC system can be described by a single order parameter defined by $\eta \equiv \left(\rho_{A}-\rho_{B}\right)/\left(\rho_{A}+\rho_{B}\right)$, $\rho_{A}$ and $\rho_{B}$ being the local monomer densities of species *A* and *B*, respectively. Note that the introduced order parameter η has a DBC-state independent volume average of −Δf. The proper thermodynamic potential that needs to be minimized to determine the local values of η by preserving its volume average is, therefore, the grand potential Ω. Ω is defined by the standard expression

(1) $$\Omega =F-\left(\rho /2\right)\int \eta \left(\mu -\Delta w\left(\vec{r}\right)\right)d^{3}r,$$

where *F* is the Helmholz free energy, $\rho =\rho_{A}+\rho_{B}$ is the spatially independent total density of the incompressible DBC system, $\mu \equiv \mu_{A}-\mu_{B}$ is the Lagrangian multiplier that enforces the above global conservation law, $\mu_{A}$ ($\mu_{B}$) being the chemical potential of the *A* (*B*) blocks. Equation (1) accounts for the presence of external potentials $w_{A}$ and $w_{B}$ acting on blocks *A* and *B*, respectively. $\Delta w\left(\vec{r}\right)\equiv w_{A}-w_{B}$ that enters the right hand side (r.h.s.) of this equation quantifies the difference between these potentials.

In order to bring the above expression for Ω into the explicit form, one must specify the form of the free energy *F*. We chose to work with the well established form of *F* based upon the celebrated Ohta-Kawasaki [45] structure factor. This form is given by

(2) $$\begin{array}{c} \beta F=-\frac{1}{2B}\int \left[\eta \left(\vec{r}\right)\nabla^{2}\eta \left(\vec{r}\right)+\xi^{-2}\eta {\left(\vec{r}\right)}^{2}\left(1-\frac{\eta {\left(\vec{r}\right)}^{2}}{2}\right)-\right.\\ \left.\frac{\lambda \xi^{-4}}{4\pi }\left(\eta \left(\vec{r}\right)+\Delta f\right)\int {\left|\vec{r}-{\vec{r}}_{1}\right|}^{-1}\left(\eta \left({\vec{r}}_{1}\right)+\Delta f\right)d^{3}r_{1}\right]d^{3}r, \end{array}$$

where $B=2NR_{G}^{-2}\rho^{-1}\left(1-\Delta f^{2}\right)$, $\beta ={\left(kT\right)}^{-1}$ is the reciprocal temperature, *k* the Boltzmann constant, *T* the absolute temperature, Δf=1−2f the asymmetry parameter, $R_{G}$ the gyration radius of copolymers, $\xi =R_{G}/\sqrt{4f(1-f)\alpha -o/(f(1-f))}$ the correlation length, and $\lambda =3\xi^{4}/R_{G}^{4}f\left(1-f\right)$. According to the Ohta-Kawasaki theory, *o* is the adjustable parameter that depends on the morphology of a DBC microphase. This parameter is described in the next paragraphs in detail.

The main advantage of the used expression for the free energy is that it properly describes the order-disorder and order-order transitions between the random phase (“melt”) and different microphases of DBC upon changing temperature. Importantly, the transition path critically depends on the DBC composition *f*. In the symmetric DBC (f=0.5), decreasing temperature below a critical value results in the direct order-disorder transition (ODT) from the random phase to the lamellar microphase. In the case of the asymmetric DBC (f≠0.5), the described transition to the lamellar microphase, caused by descreasing temperature, occurs by crossing through intermediate microphases having reduced symmetry (e.g., cylindrical, spherical, gyroid).

An important adjustment of the original Ohta-Kawasaki theory to more recent results, described below, is in order here. In fact, the described transition between different DBC microphases at a given composition *f* is fully controlled by segregation parameter α that enters the definition of correlation length ξ given below Equation (2). The critical value of the segregation parameter, $\alpha_{c}\left(f\right)$, corresponding to the ODT transition can be directly related to the adjustable parameter *o* introduced in the definition of ξ. This relation can be deduced from the condition that the Ohta-Kawasaki structure factor of random DBC phase must not diverge. This restriction, along with the spinodal condition for DBC, determines a simple relationship between $\alpha_{c}$ and *o* of the form $\alpha_{c}={\left(2f\left(1-f\right)\right)}^{-2}o+\sqrt{0.75{\left(f\left(1-f\right)\right)}^{-3}}$. It is straightforward to show that the above relation gives o=0.9 used in the Ohta-Kawasaki theory for $\alpha_{c}\approx 10.5$ predicted by the seminal work of Leibler [31] for the case of the symmetric DBC. Taking into account the fluctuation effects [47] results in a significant correction to the described Leibler result. According to the FTS theory [48], $\alpha_{c}∼20$ for the above symmetric case. To incorporate the described correction to $\alpha_{c}$, we choose to work with parameter $\Delta \alpha \equiv \alpha -\alpha_{c}$ quantifying the departure from the critical point in the DBC phase diagram. In the present theory, $\alpha_{c}$ is therefore treated as an external parameter that can be obtained directly from experiments. It proves to be more convenient to use control parameter λ defined right below Equation (2) to track the microphase separation of DBC. This parameter can be straightforwardly expressed in terms of Δα as follows

(3) $$\lambda =3{\left(4{\left(f\left(1-f\right)\right)}^{\frac{3}{2}}\Delta \alpha +2\sqrt{3}\right)}^{-2}.$$

The Euler-Lagrange equation for η can be obtained by the minimization of Ω. This equation reads

(4) $$\nabla^{2}\eta \left(\vec{r}\right)+\xi^{-2}\eta \left(\vec{r}\right)\left(1-\eta {\left(\vec{r}\right)}^{2}\right)-\frac{\lambda }{4\pi }\int {\left|\vec{r}-{\vec{r}}_{1}\right|}^{-1}\left(\eta \left({\vec{r}}_{1}\right)+\Delta f\right)d^{3}r_{1}=-B\rho \beta \left(\mu -\Delta w\right)/2.$$

In the absence of the external potential (Δw=0), the form of the solution of Equation (4) is fully determined by control parameter λ. Specifically, for λ<0.25, Equation (4) has periodic solutions describing the misrophase-separated DBC. For λ>0.25 Equation (4) has the only zero solution corresponding to the random DBC phase. The described periodic solutions $\eta_{eq}\left(\vec{r}\right)$ of Equation (4) were obtained numerically by employing the accurate isogeometric finite element method developed in [49]. This method makes it possible to obtain the DBC morphologies used for the prediction of the localization of fillers, as described in Section 2.4.

Interestingly, the obtained Equation (4) can be directly used to derive the expression for the equilibrium grand potential Ω. Excluding the gradient terms in Equation (2), one arrives at

(5) $$\Omega =-{\left(4\beta B\xi^{2}\right)}^{-1}\int \eta_{eq}{\left(\vec{r}\right)}^{4}d^{3}r+4^{-1}\rho \int \eta_{eq}\left(\vec{r}\right)\Delta w\left(\vec{r}\right)d^{3}r,$$

where $\eta_{eq}\left(\vec{r}\right)$ is the equilibrium order parameter defined as the solution of Equation (4). The obtained expression is directly used in Section 2.3 to explicitly derive the filler immersion energy.

### 2.3. Filled DBC System: Immersion Energy of Fillers

Consider a spherical hard filler having radius *R* added to the DBC system described in Section 2.2. We assume that this filler has different affinities for dissimilar DBC blocks. As is shown in what follows, this affinity difference promotes the selective localization of fillers in the DBC microphases. Note that Equation (4) derived in Section 2.2 is perfectly suited to incorporate the effect of fillers on the DBC morphological structure. This effect can be described by specifying potentials $w_{A,B}$ in the last term in the r.h.s. of this equation. To quantify the affinity of copolymer blocks for fillers, we set the following form of these potentials describing the polymer-filler interactions

(6) $$w_{A,B}=-\rho^{-1}\gamma_{A,B}\delta \left(r-R\right),$$

where δ(r) is the Dirac delta function, *r* is the distance to the filler center, and $\gamma_{A,B}$ is the adhesion energy per unit area, hereafter referred to as “adhesion”. It is important to note that we restrict our consideration to the weak reversible adsorption of polymers onto fillers described by the above potentials $w_{A,B}$ having infinitesimally small range. The advantage of the employed simple form of $w_{A,B}$ given by Equation (6) is twofold. Firstly, adhesion $\gamma_{A,B}$ can be measured experimentally by the Wilhelmy method [21,23], thus providing solid relation between the proposed theory and experiment. Secondly, the form of $w_{A,B}$ makes it reasonable to assume that the considered weak adsorption interactions do not significantly affect the structure of the incompressible DBC in the vicinity of fillers. This assumption is justified by both the experiment [50,51,52,53] and simulations [54] for the considered case of small ratios of the filler diameter to the typical DBC domain size, relatively small filler volume fractions, and weak attraction between fillers.

The immersion energy *W* of a filler is defined [21,22,55,56] as a minimal work required to place this filler in the DBC system while maintaining given thermodynamic conditions. Mathematically, this energy is expressed as a difference of the grand potential given by Equation (7) in the presence of a filler and its filler-free counterpart. Using the explicit expression for the adhesion potentials given by Equation (6), *W* reduces to

(7) $$\beta W=-{\left(8\pi \right)}^{-1}\sigma \int \eta_{eq}\left(\vec{r}+R\vec{n}\right)d\vec{n}+{\left(4B\xi^{2}\right)}^{-1}\int_{V_{R}}\eta_{eq}{\left(\vec{r}\right)}^{4}d^{3}r,$$

where we have introduced the dimensionless parameter $\sigma =2\pi R^{2}\beta \gamma$ ($\gamma =\gamma_{A}-\gamma_{B}$) hereafter termed as the affinity contrast, $\vec{n}$ is the unit vector directed along the positive normal to the filler surface.

Note that the integration in the first term in the r.h.s. of Equation (7) is over the filler surface area, while the integration in the second term is over the volume $V_{R}$ of a filler. The mathematical structure of the described terms stems from the fact that the first term describes the interaction between the surface of a filler and DBC, while the second term describes the excluded volume (osmotic) effect. The first surface term is directly proportional to affinity contrast σ. This term therefore promotes the localization of a filler in the DBC microphase having larger affinity for this filler. The second volume term describes the excess enthalpy of DBCs expelled from the volume occupied by a filler. This term is, therefore, of the osmotic nature. In contrast to the first surface term, the described volume term favors the localization of fillers at the interfaces between the DBC microphases. In this position, these fillers prevent energetically unfavourable contacts between dissimilar DBC blocks. As the relative significance of the described terms essentially depends on the location of a filler in the DBC system, the calculated immersion energy is directly relevant to the probability to find a filler in a given location in this system. We use this fact to determine the equilibrium localization of fillers in DBC, as described in the next section.

### 2.4. Localization of Fillers in DBC System Predicted by Monte-Carlo Simulations

According to the Widom principle [57] the obtained immersion energy *W* (excess chemical potential caused by the presence of a filler) determines the probability to find a filler in a given location in the DBC system in the absence of other fillers. For the considered dilute solution of fillers in DBC, this energy gives the main contribution to the Bolzmann factor determining the probability of a given filler localization. With increasing the filler concentration, an additional contribution to the system energy, caused by the interaction among fillers, comes into play. Note that in addition to the conventional molecular (e.g., van der Waals) interactions, one must also take into account the effective interactions [58,59,60] between fillers mediated by a polymer system having non-uniform density or composition. In the considered case of weakly adsorbing fillers and incompressible DBC, the polymer-mediated effective interactions are negligible relative to the molecular interactions. Based on this observation, in the present work we model the total interaction between fillers by the position-independent potential *U* that encompasses all the pair interactions acting in the filler system.

The total energy of the filler system $E=\sum_{i,j}\left(W_{i}\delta_{i}^{j}+U_{ij}\right)$, where the indices i,j enumerate the filler particles, can be obtained for each spatial distribution of fillers in DBC. This energy is used in the standard lattice Metropolis Monte-Carlo simulation algorithm [61] to determine the localization of fillers in a DBC system at equilibrium. Recall that in the case of compositionally non-uniform DBC morphologies caused by the microphase separation, *E* depends on the location of fillers in the DBC system. The above simulation procedure, therefore, elucidates the relation between a specific morphology of the DBC system and the most probable localization of fillers in this system.

The performed thorough simulations show that the localization of fillers in DBC depends on the main four factors, as follows: (i) the difference between the affinities of fillers for dissimilar DBC blocks; (ii) strength and sign of the interaction between fillers; (iii) temperature of the DBC system and (iv) geometry of the DBC morphology determined by the temperature (segregation α) and DBC composition *f*. The detailed account of the effects of the above factors on the localization of fillers in DBC is given in the corresponding subsections of Section 3.2.2.

### 2.5. Calculation of the Conductivity of a Filled DBC System

The DBC morphology-driven localization of fillers, described in Section 2.4, can result in the formation of a percolating filler network that conducts electrical current. This current, conducted by the fillers, is caused by a constant voltage *u* imposed across a layer of the filled DBC system. We consider that *A*- and *B*-microphase domains of the separated DBC are oriented perpendicularly to the electrodes confining this layer. This lamellar orientation makes it possible to better elucidate the conductor-insulator transition described in Section 3.2.4. The DBC are considered totally insulating, while the fillers are highly conductive.

The outcome of the lattice Monte-Carlo simulations described in Section 2.4 is used to calculate the conductivity of the considered filled DBC system. Note that for the insulating DBC system under consideration, the conductivity of the composite is determined by the conductive filler network formed within this composite. Fillers can form a percolating conductive network consisting of continuous clusters of fillers that make close contact. The theoretical development can be thus greatly facilitated by representing the filler system formed in DBC as a regular resistor network, as described below.

The performed calculation focuses on the main quantity of interest, the reduced resistivity *r*. This quantity is defined as the ratio of the resistance $r_{C}$ of the composite to the resistance $r_{0}$ of a randomly loosely packed pure filler system of the same size. The value of $r_{C}$ is determined for a given filler volume fraction ϕ by computing the current through the resistor network formed by the fillers. $r_{0}$, required to evaluate ratio $r=r_{C}/r_{0}$, is calculated within the same resistor network model for the lattice fully occupied by fillers. The calculated *r* is then used to obtain the composite resistivity in terms of that of a pure filler material, known for most of the fillers used in practice.

The described resistor network is constructed on the basis of a cubic lattice with a period equal to the filler diameter 2R. In each simulation run, the centers of *M* fillers are randomly placed in the vertices of the lattice. The bonds of the lattice connecting two vertices occupied by the filler centers are treated as resistors having resistance $r_{1}$. The remaining bonds are assigned infinite resistance (zero conductance). An important remark on the choice of the lattice size is in order here. As the lattice period is fixed and the period of a DBC morphology can vary with varying Δα, the number of bonds *L* in each lattice direction varies depending on the size of DBC microphases. We, therefore, express 2R and *L* through the copolymer gyration radius $R_{G}$ that is used as a length scale naturally arising from the DBC morphological structure. *L* is therefore obtained for each simulation run by adjusting to the DBC morphology obtained in this run.

**Figure 1:**
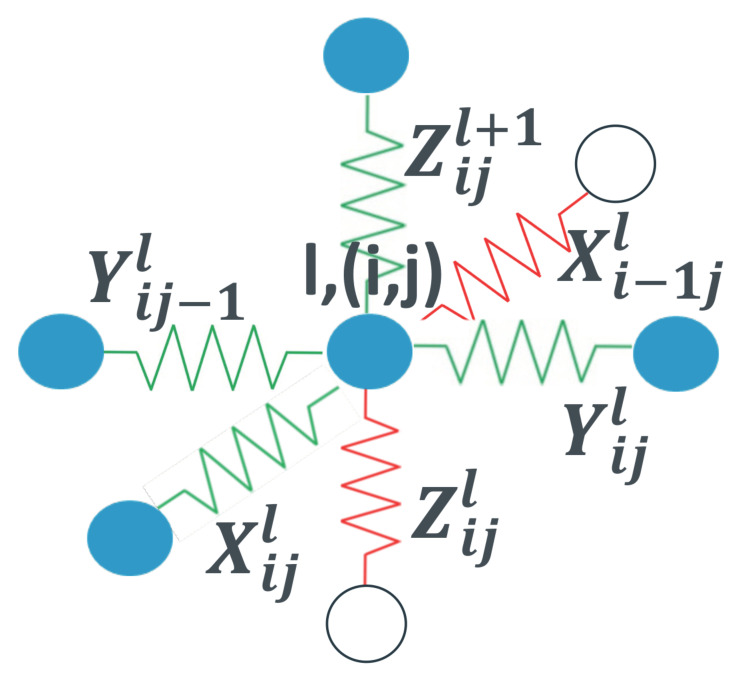
Unit cell of the constructed random resistor network. See the explanation and notations in the text.

To formalize the above filler network model construction, we enumerate the position of the vertices of the described cubic resistor network by integer *l* and tuple (i,j) (see Figure 1). Here, *l* enumerates the lattice layers parallel to the electrodes, counting from the negative electrode numbered l=0. *i* and *j* enumerate the abscissa and ordinate indexes of each vertex in layer *l*, respectively. The three mutually perpendicular bonds having one common end in vertex l,(i,j) are denoted by $X_{i,j}^{l}$, $Y_{i,j}^{l}$ and $Z_{i,j}^{l}$. Here, $Z_{i,j}^{l}$ is normal to the electrodes and $X_{i,j}^{l}$, $Y_{i,j}^{l}$ are parallel to them. The described normal bonds form a basis of the unit cell of the Bravais cubic lattice.

The described lattice construction makes it possible to directly apply the Kirchhoff’s relations to each vertex l,(i,j) of the resistor network. These relations read as

(8) $$\begin{array}{c} V_{i,j}^{l+1}=V_{i,j}^{l}+Z_{i,j}^{l}I_{i,j}^{l+1},\\ I_{i,j}^{l+1}=I_{i,j}^{l}+X_{i,j}^{l}\left(V_{i,j}^{l}-V_{i+1,j}^{l}\right)+X_{i-1,j}^{l}\left(V_{i,j}^{l}-V_{i-1,j}^{l}\right)+\\ Y_{i,j}^{l}\left(V_{i,j}^{l}-V_{i,j+1}^{l}\right)+Y_{i,j-1}^{l}\left(V_{i,j}^{l}-V_{i,j-1}^{l}\right), \end{array}$$

where $I_{i,j}^{l}$ and $V_{i,j}^{l}$ are the current and voltage at vertex l(i,j).

The first equality in Equation (8) give the iteration relations between the currents and voltages in the adjacent layers l+1 and *l*, while the second equality describes the current flow in each layer. These relations are solved numerically in the presence of the boundary conditions expressing the restriction that the electrodes are maintained at constant voltages. These conditions are imposed on layers 0 and *L* and read $V_{ij}^{0}=0$ and $V_{ij}^{L}=u$ for these layers, respectively. By making use of the obtained solution, the currents in all bonds are derived for each distribution of fillers in the lattice obtained by the Monte Carlo simulations described in Section 2.4.

Using the described solution for the bond currents, we calculated resistance $r_{C}$ of the resistor network by directly employing the Ohm’s law $r_{C}=u/\sum_{i,j}\left(I_{i,j}^{L}-I_{i,j}^{0}\right)$. Note that the ratio $r_{C}/r_{1}$ of the network resistance to the elementary resistance is $r_{1}$-independent, as all the non-zero currents $I_{i,j}^{L}$ are proportional to $1/r_{1}$. The desirable reduced resistivity $r_{C}/r_{0}$ of the composite for a selected DBC morphology and filler localization is, therefore, given by a filler material-independent constant that is less than unity. The absolute, filler material-specific resistivity of the composite, can be calculated by multiplying the obtained reduced resistivity by the resistivity of a filler material that is considered a given external parameter of the theory.

## 3. Main Simulation Results and Discussion

### 3.1. Elementary Test of the Phase-Field Model for an Unfilled DBC System

**Figure 2:**
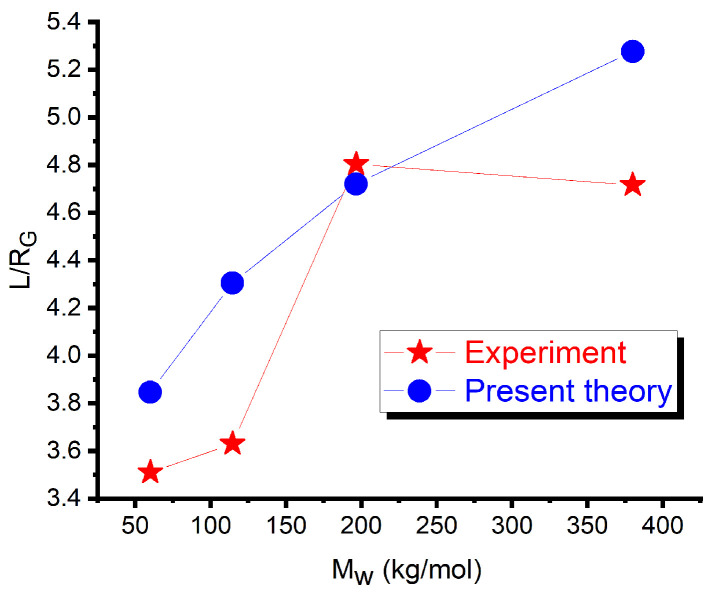
Comparison of the reduced lamella period calculated by numerically solving the phase-field Equation (4) with Δw=0 against the experiment in [36]. All lengths are measured in the gyration radii of the DBC derived from their molecular weights assuming the monomer length of 0.5 nm. Adapted with permission from [27], APS, 2020.

To demonstrate the applicability of the applied numerical finite element method to solving the phase-field equations described in Section 2.2, we tested this method for the simplest case of the unfilled symmetric DBC, well documented experimentally. As a test case, we used the optical observations of the morphological structure of the symmetric DBC polystyrene (PS)-b-2 vinylpyridine (P2VP) having four different molecular weights $M_{w}$, reported in [40]. To mimic the experimental conditions, we solved Equation (4) with f=0.5 and the value of λ calculated from Equation (3) with χ=0.178 [52]. The polymerization degrees *N* used in Equation (3) are derived from the molecular weights of the DBC used in the respective cases studied in the experiment. Figure 2 shows the comparison of the lamella period, derived from the obtained finite element solution of Equation (4) with Δw=0, against its experimentally measured counterpart. This figure demonstrates that the used numerical method gives an adequate estimate for all the DBC molecular weights covering the range from the intermediate segregation regime (α=102 for $M_{w}=60$ kg/mol) to the strong segregation one (α=645 for $M_{w}=380$ kg/mol).

The present comparison shows that the used finite element method gives adequate predictions for the microphase structure of the DBC system.

### 3.2. Localization of Fillers in DBC System

#### 3.2.1. Effect of the Filler Affinity Contrast

**Figure 3:**
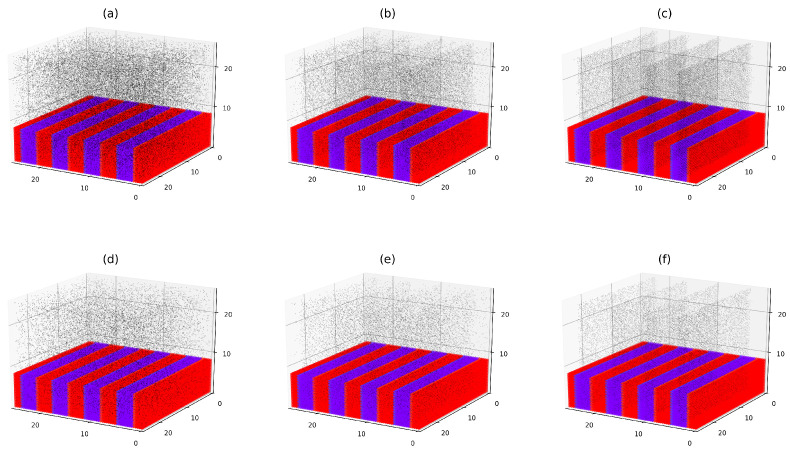
Effect of the difference in affinities of fillers for dissimilar copolymer blocks on their localization in the DBC lamellae. Filler volume fractions ϕ for plots shown in the upper and lower panels are set to 0.078 and 0.037, respectively. For each ϕ, several cases corresponding to selected values of affinity contrast, σ, are shown: ϕ=0.078: (**a**) σ=0.0, (**b**) σ=5.3, (**c**) σ=133.1; ϕ=0.037: (**d**) σ=0.0, (**e**) σ=5.3, (**f**) σ=133.1. Black dots represent the centers of the filler particles. The upper part of the images of the host DBC is cut off to make the localization of fillers inside the DBC system visible. The selective *A* phase, which has a larger affinity for polymers, is shown in red, and the *B* phase is shown in violet. All lengths are measured in $R_{G}$.

Thorough simulations of the filled symmetric diblock copolymer system have been performed as is described in Section 2.3 and Section 2.4, for selected values of the affinity contrast σ and the reduced inter-filler potential βU. An illustrative example of these simulations for βU=1 and varying σ is shown in Figure 3. The chosen value of the interaction potential corresponds to a weak repulsion between fillers that slightly suppresses their crowding upon close approach. The reduced segregation parameter Δα is set to 55, which corresponds to relatively strong segregation of DBCs. For this value of Δα, the symmetric DBC system consists of alternating, well geometrically defined lamellar layers of the *A*-rich and *B*-rich microphases (see Figure 3). The radius of fillers is set to $R=0.1R_{G}$.

For each filler volume fraction considered in the simulations, the affinity contrast σ is incrementally increased from 0 to 300. The results of these simulations, for selected values of σ, are illustrated in the upper and lower panels of Figure 3, corresponding to filler volume fractions ϕ=0.078 and ϕ=0.037, respectively. As is clearly seen from Figure 3, the value of σ plays a critical role in tailoring the localization of fillers in the DBC system. For the case of relatively small values of σ, illustrated in Figure 3a,d, the fillers are diffusely distributed throughout the DBC system. For the case of moderate values of σ, illustrated in Figure 3b,e, the fillers tend to localize primarily within the selective *A*-phase, with some accumulation at the interfaces. At large values of σ, the fillers are preferentially localized in the selective *A*-phase (see Figure 3c,f).

The preferential localization of fillers within the selective microphases of the DBC, as observed in Figure 3c,f, is a direct consequence of their higher affinity for the *A*-microphase, quantified by the parameter σ. It is important to note that in these cases the surface interactions, corresponding to the larger values of σ>∼10, dominate over the osmotic (excluded volume) interactions, described by the second volume term in the r.h.s. of Equation (7). The described dominance of the surface interactions arises from the fact that the surface and osmotic contribution to the filler immersion energy are of the order of $\eta_{m}$ and $\eta_{m}^{4}$, respectively. Here, $\eta_{m}$ denotes the maximum value of the order parameter. For the considered case of Δα=55, we have $\eta_{m}∼0.5$ and $\eta_{m}^{4}∼0.05$, highlighting the relatively minor role of the osmotic effect. This fact explains the less pronounced tendency to the interface filler localization observed in Figure 3c,f.

To verify our theoretical findings and further elucidate the competition between the above described surface and osmotic effects, we compared our simulation results against the experimental results in [52]. This study investigates the localization of gold (Au) fillers functionalized with a binary mixture of thiol end-functional polystyrene (PS) ligands and poly(2-vinylpyridine) (P2VP) homopolymer ligands, both of low molecular weight. A symmetric PS-b-P2VP DBC is employed as the host polymer system.

It is important to note that the used gold fillers have stronger affinity for the P2VP blocks. The relative affinity of the fillers for the copolymer blocks is tuned by varying the surface fraction $F_{PS}$ of PS ligands grafted onto the filler surface. The localization of the thus modified fillers in the PS-b-P2VP lamellar domains, depending on $F_{PS}$, has been studied by the transmission electron microscopy. At high $F_{PS}$ values, the fillers were found to be predominantly dispersed near the center of the PS domains. As $F_{PS}$ was decreased below a critical surface fraction, $F_{PS}^{c}∼0.8$, the fillers exhibited a tendency to segregate toward the PS/P2VP interface, and in some cases, even partially relocated into the P2VP microphases.

**Figure 4:**
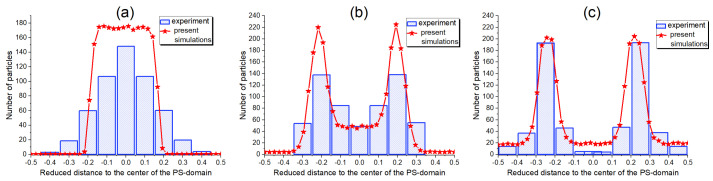
The localization of gold fillers in the lamellar domains of the DBC PS-b-P2VP for several selected values of the surface fraction $F_{PS}$ of the PS-ligands: (**a**) 0.92, (**b**) 0.90, and (**c**) 0.80. The interface boundaries of the PS domain are located at −0.25 and 0.25. The experimental histograms in blue show the filler localization in the cross section of the lamellar domains, as obtained in [52] from transmission electron microscopy micrographs. The line-symbol curves in red show the simulation results for the values of the affinity parameter *g*, evaluated for the corresponding $F_{PS}$. See the explanation in the text. Adapted with permission from [27], APS, 2020.

The results of our simulations that mimic the above described experiment, are depicted in Figure 4. The polymerization degree is set to N=1875, corresponding to the molecular weight $M_{w}=196.5$ kg/mol of the PS-b-P2VP DBC used in the experiment. The PS-b-P2VP Flory-Huggins parameter is set equal to its experimentally known [52] value of 0.178 (at 25 °C). The filler particle radius is set to R=2.5 nm, consistent with the experimental conditions, and the total number of particles to 60,000, in order to match the experimental filler fraction of 0.15 used in [40,52]. In our simulations, the affinity contrast is quantified by the dimensionless affinity parameter $g={\left(4\pi \right)}^{-1}b^{2}\sigma$ evaluated from the experimental conditions, where *b* is the monomer length. *g* was shown [27] to be related to the surface fraction $F_{PS}$ by $g\equiv 2\beta \gamma_{PS-PVP}b^{2}F_{PS}\left(1-F_{PS}\right)$, where $\gamma_{PS-PVP}$ is the difference between the affinities of the PS and P2VP blocks for the gold fillers. Under the given experimental conditions, $\gamma_{PS-P2VP}$ is estimated to be $12\;{\mathrm{mJ}/m}^{2}$. Given this value of $\gamma_{PS-P2VP}$, the affinity parameter *g* evaluates to 0.020, 0.014, and −0.005 for the experimental PS surface fractions 0.92, 0.90, and 0.80, respectively.

A comparison between the simulated spatial distribution of fillers and the corresponding experimental results is presented in Figure 4, based on the set of parameters derived from the experimental conditions described above. To allow direct comparison with the experiment, the simulated filler distribution is averaged over all directions except the one perpendicular to the lamellae. The simulations predict three distinct types of filler localization, as shown in the corresponding panels of Figure 4: (a) at $F_{PS}=0.92$, the fillers are predominantly dispersed near the center of the PS domain; (b) at $F_{PS}=0.90$, the fillers are mainly segregated to the interfaces between the PS and P2VP blocks; (c) at $F_{PS}=0.80$, a fraction of the fillers is localized at the interface, while the remainder is distributed within the P2VP phase.

As shown in Figure 4, the simulations provide an accurate prediction of all experimentally observed filler density profiles. Particularly good agreement is observed for cases (b) and (c), corresponding to the moderate and low PS-ligand surface fractions of 0.80 and 0.90, respectively. In case (a), which corresponds to the highest PS-ligand fraction ($F_{PS}=0.92$), the simulations slightly overestimate the local concentration of fillers within the PS microphases. In this case, the fillers have the largest affinity for the PS-blocks and, therefore, tend to localize within the PS-microphases. It is important to emphasize that the simulation results shown in Figure 4 are obtained without any adjustable parameters. Adjusting the value of the affinity parameter *g*, performed in [27], is shown to further improve the agreement between the present theory and the simulations.

The above described analysis of our simulation results illustrated in Figure 4, therefore, leads to a qualitative conclusion that the affinity contrast σ is the decisive factor determining the localization of fillers in the DBC system, particularly when inter-particle interactions are relatively weak (U⪅kT). When σ is sufficiently large for surface interactions to dominate over osmotic effects, the fillers preferentially localize within the selective *A*-domains (see case (a) in Figure 4). For intermediate values of σ, where surface and osmotic contributions are comparable, the fillers are distributed across broader regions, including both the *A*-microphases and the interfaces between the microphases *A* and *B*. Finally, than the osmotic effect prevails, the fillers are segregated to the interfaces between the microphases (see Figure 4c). Interestingly, this qualitative behavior is found to be only weakly dependent on the total volume fraction of fillers.

#### 3.2.2. Effect of the Interaction Between Fillers

For filler volume fractions of the order of 0.05 and higher, the simulations reveal a significant influence of inter-particle interactions on filler localization. In the present approach, the interactions between fillers consist of two components: short-range steric (excluded volume) interactions and molecular interactions (e.g., van der Waals forces). Steric interactions are inherently accounted for by enforcing unique occupancy of lattice sites in the Monte Carlo simulations. Molecular interactions, on the other hand, are represented by the total interaction potential *U* as described in Section 2.4. In the simulations, we explore both attractive and repulsive inter-particle interactions, with interaction strengths varying within the range −10kT<U<10kT.

**Figure 5:**
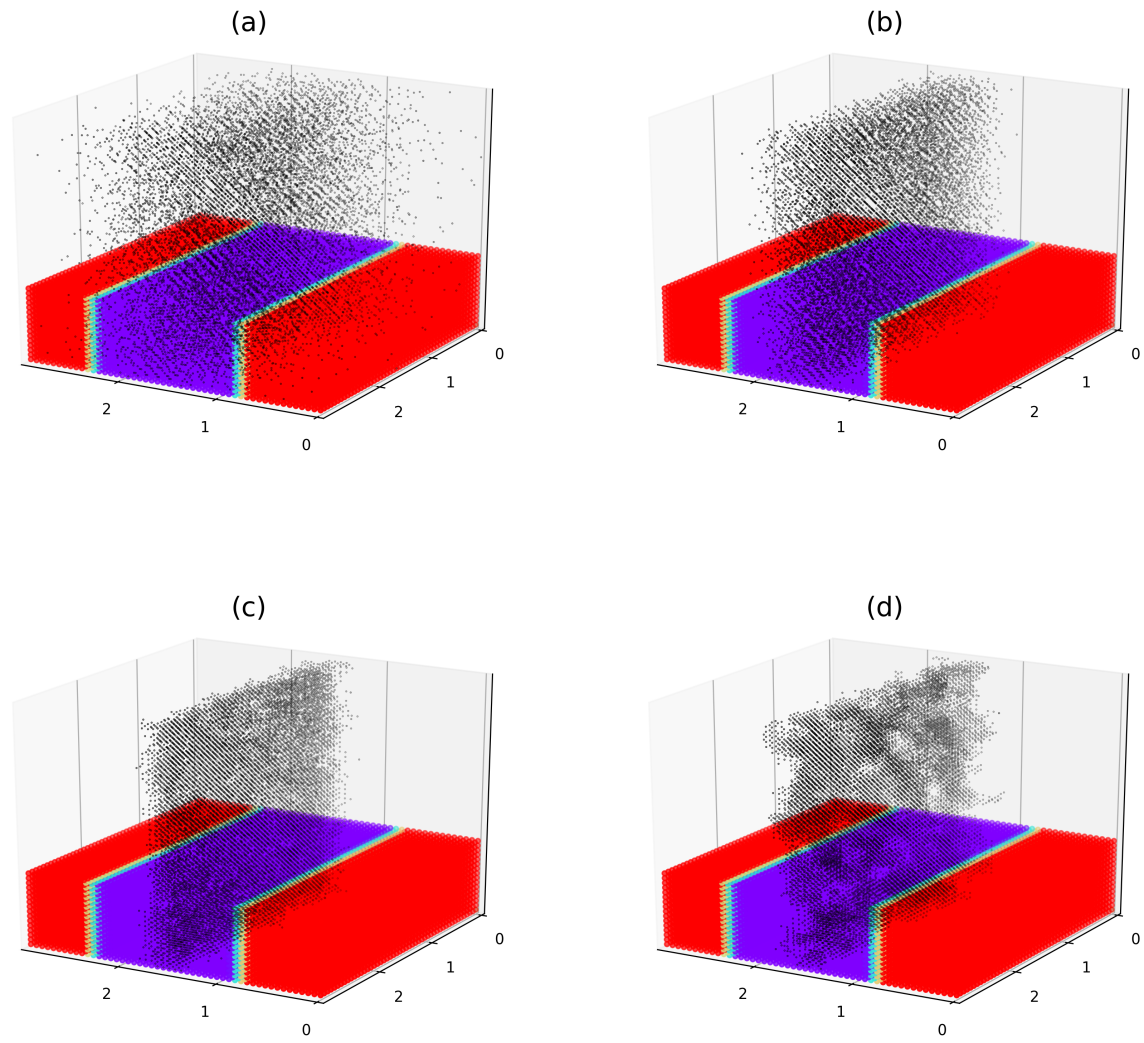
Localization of fillers, interacting through the potential *U*, in the lamellae formed by the microphase-separated DBC system. The energy of the inter-particle interaction *U* is set to: (**a**) 10.0kT; (**b**) 1.0kT; (**c**) −1.0kT; (**d**) −10.0kT. The reduced segregation parameter Δα is set to 12.1. Black dots represent the centers of the filler particles. The upper part of the image of the host DBC matrix is cut off to make the distribution of fillers inside the DBC system visible. The selective *A* phase, which has a larger affinity for fillers, is shown in red, while the *B* phase is shown in violet. All lengths are measured in $R_{G}$. The radius of the fillers is $0.07R_{G}$. The volume fraction of fillers is 0.052.

Typical examples of the simulated filler distributions for different values of *U* and fixed σ are presented in Figure 5. This figure illustrates the spatial distribution of filler particles for several values of the reduced interaction potential βU. The reduced segregation parameter Δα, which determines the morphology of the DBC, is set to 12.1, corresponding to a moderately segregated regime. The filler volume fraction is fixed at ϕ=0.052, and the affinity contrast is set to σ=5.3. This combination of parameters corresponds to the case, when the percolation transition in the filler network occurs at βU=−0.1 for the specified values of ϕ and Δα. For the specified parameter values, in the absence of inter-filler interactions (i.e., βU=0), the fillers show a slight tendency to localize within the selective *A*-microphases shown in red in Figure 5.

As can be elucidated from Figure 5a, the fillers experiencing sufficiently strong repulsion U∼5kT are diffusely distributed throughout the DBC system. In this case, the repulsive inter-particle interactions override the previously described tendency toward selective localization driven by the filler preferential affinity for one of the copolymer blocks. In contrast, relatively weak inter-particle interactions, whether repulsive or attractive, do not significantly influence the spatial distribution of the fillers, as demonstrated in Figure 5b,c. However, when the interaction becomes strongly attractive (U∼−5kT), it reinforces the effect of the affinity contrast, thereby enhancing the selective localization of fillers within the attractive microphases. As can be seen in Figure 5d, this effect also results in additional clustering of fillers that lowers the percolation threshold in the filler network. This clustering, in turn, facilitates current percolation through the filler network, as is discussed in more detail in the Section 3.3.

It should be noted that the enhanced clustering of fillers observed at relatively strong attractive interactions emerges only when the local filler concentration is sufficiently high. This behavior is attributed to the fact that short-range attractive forces contribute significantly to the total energy of the filler system only when average interparticle distances are small. Such close spacing of fillers, particularly within the *A*-microphases, occurs only when there is a substantial difference in the affinities of the fillers for the copolymer blocks. Consequently, in cases of weak to moderate inter-filler interactions, the effect on filler localization remains minor if the affinity contrast is low. Thus, the affinity contrast primarily governs filler localization in the DBC system, especially at low filler volume fractions (ϕ≤0.05). This effect may be further amplified or diminished by sufficiently strong attractive or repulsive interactions between fillers, respectively.

#### 3.2.3. Effect of the DBC Morphology

**Figure 6:**
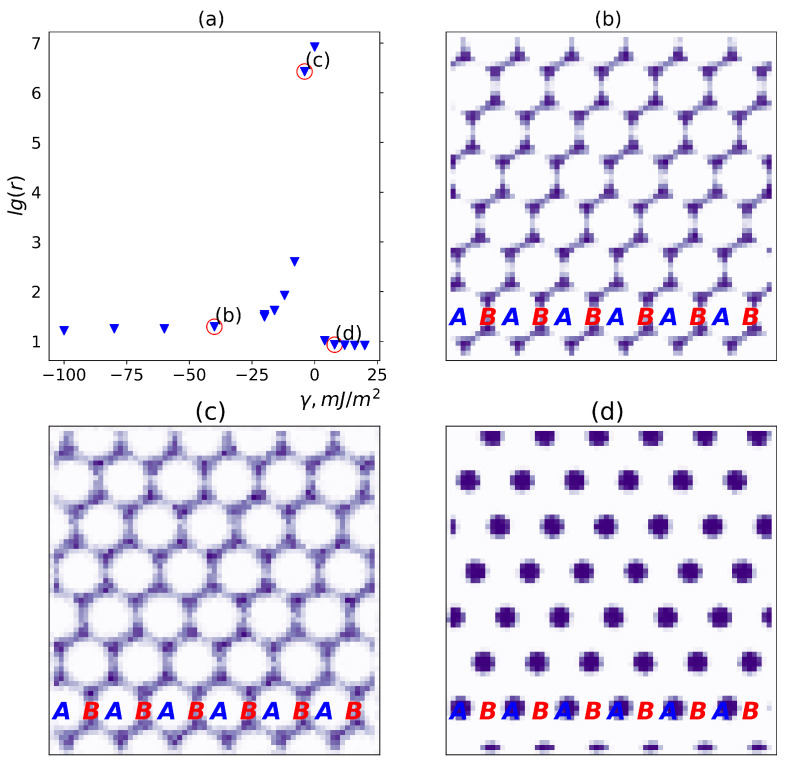
Effect of difference γ between affinities of fillers for dissimilar copolymer blocks on the conductivity of the microphase-separated asymmetric DBC system (f=0.45) and the localization of fillers in this system. The reduced segregation Δα, volume fraction of fillers ϕ, and reduced inter-filler interaction energy βU are set to 32.6, 0.078, and −1.0, respectively. (**a**) Conductivity as a function of γ. Red circles highlight the points corresponding to the cases of the respective γ values illustrated in subplots (**b**–**d**). (**b**–**d**) Localization of fillers for selected values of γ: (**b**) $\gamma =-40\;{\mathrm{mJ}/m}^{2}$, (**c**) $\gamma =-4\;{\mathrm{mJ}/m}^{2}$, (**d**) $\gamma =8\;{\mathrm{mJ}/m}^{2}$. See the explanation in the text.

To study the effect of the DBC microphase morphology on the localization of fillers in relation to the effects of the affinity contrast and inter-filler interactions described in Section 3.2.1 and Section 3.2.2, respectively, it is instructive to examine the case of the asymmetric DBC with f=0.45. In the performed Monte-Carlo simulations we used the DBC morphology obtained from the solution of Equation (4) for Δα=32.6 that lies above the order-order transition from the lamellar to cylindrical DBC morphology. The results obtained in the simulations for the filler volume fraction ϕ=0.078 are illustrated in Figure 6. This figure demonstrates that the localization of fillers can be effectively altered by changing the affinity contrast γ at a given inter-filler interaction energy *U*.

For the used values of *f* and Δα, the DBC system is shown to separate into the *A*-rich minority cylindrical (hexagonal) phase and the *B*-rich majority phase. The simulations have been performed for several values of γ quantifying the affinity contrast of the fillers for dissimilar copolymer blocks. Subplots (b)–(d) in Figure 6 show the local concentration of fillers averaged over the directions perpendicular to the cylinders of the hexagonal phase. In the case (d), where γ=8mJ/m^2^ is positive, the fillers are found to be localized within the cylinders of the *A*-microphase, while in cases (b), (c) corresponding to negative values of γ, they are localized in the majority *B*-microphase.

For small affinity contrasts |γ|, illustrated in Figure 6c, i.e., in the absence of a strong affinity contrast of fillers for the copolymer blocks, the osmotic effect becomes more significant. In this case, a part of the fillers is found to be localized at the interfaces where they screen unfavorable interactions between the DBC blocks *A* and *B*. As a result, the fillers tend to be more diffusely distributed across the larger domains composed of the selective *A*-microphases and the *A*-*B* interfaces. This behavior suppresses the formation of sufficiently large filler clusters needed to establish composite conductivity, as discussed in detail in Section 3.3.

#### 3.2.4. Effect Caused by Changing the Composite Temperature

The effect of temperature on the localization of fillers observed in the simulations is twofold. Firstly, decreasing the temperature below the ODT point leads to the microphase separation in the DBC system. Further lowering the temperature in the case of the asymmetric DBC induces an order-order transition between different DBC microphases. Both effects significantly influence filler localization.

At temperatures near the ODT, the system enters the weak segregation regime characterized by relatively wide interfaces between the microphases. At temperatures far from the ODT, the system reaches the strong segregation regime with narrow interfaces between domains that contain almost pure *A*- and *B*-blocks. Since fillers tend to localize at the interfaces (due to the osmotic effect) or in the selective microphases (due to the surface effect), the morphological state of the DBC system critically affects the localization of the fillers. Secondly, temperature changes directly affect filler localization by altering the relative role of the filler translational entropy, which favors a more diffuse distribution of fillers throughout the DBC host system.

To elucidate the above temperature-induced effects, it is convenient to quantify temperature *T* of the composite in terms of the above introduced reduced segregation parameter Δα, whose enthalpic part is inversely proportional to *T*. In the simulations described in the present section, Δα serves as the sole temperature-dependent control parameter that determines the morphology of the DBC system for any given DBC composition *f*. Recall that when Δα>0, the DBC microphases form, with the degree of segregation increasing as Δα increases.

**Figure 7:**
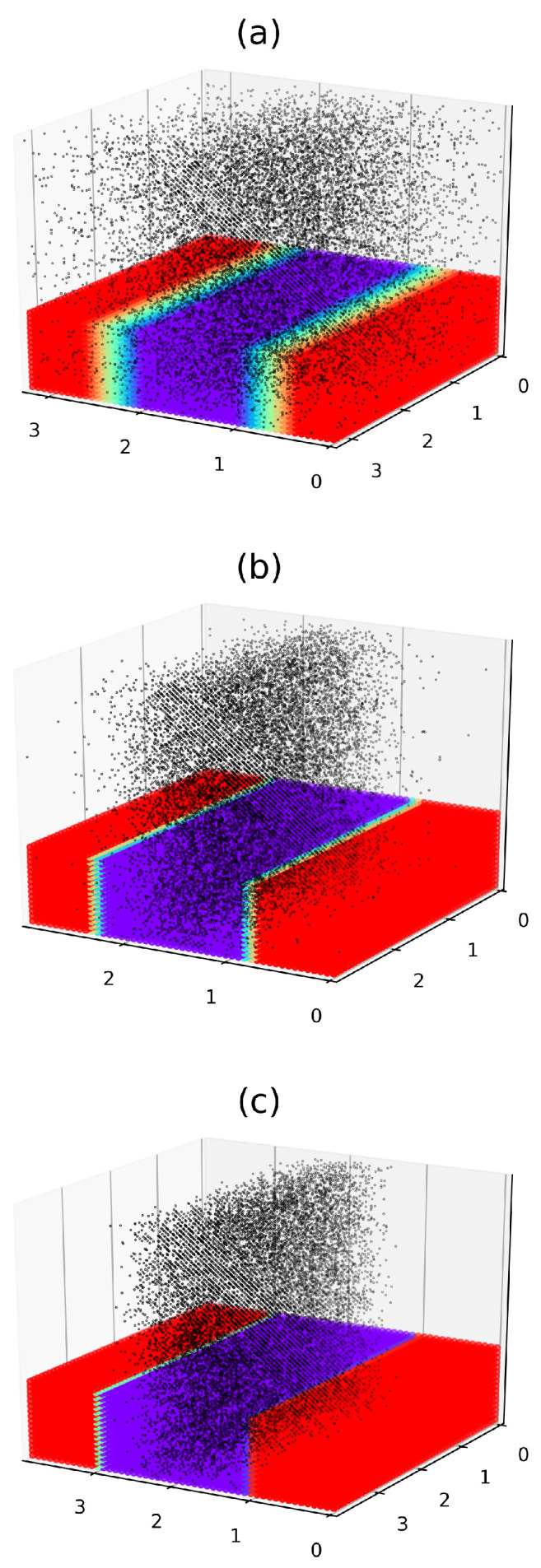
Effect of changing the degree of segregation of the DBC blocks, quantified by the reduced segregation parameter Δα, on the localization of fillers in the microphase-separated DBC system. Δα is set to: (**a**) 0.2; (**b**) 1.5; (**c**) 8.6. The centers of the filler particles are represented by black dots. The upper part of the image of the host DBC matrix is cut off to make the localization of fillers inside the DBC system visible. The selective *A* phase, which has a larger affinity for fillers, is shown in red, and the *B* phase is shown in violet. All lengths are measured in $R_{G}$, so the radius of the fillers is 0.07. The energy of the inter-particle interaction *U* is set to −0.1kT. The volume fraction of particles is 0.052.

Figure 7 shows the morphologies of the described filled symmetric (f=0.5) DBC system for selected values of Δα=0.2, 1.5, 8.6. The volume fraction of fillers ϕ and the affinity of fillers for *A* copolymer blocks are set equal to 0.052 and $12\;{\mathrm{mJ}/m}^{2}$, respectively. The interaction energy between neighboring fillers, *U*, is set to −0.1kT, corresponding to a weak attraction between closely spaced fillers. This interaction consists of van der Waals forces and short-ranged, polymer-mediated depletion interactions. Note that the used weakly attractive potential promotes the crowding of fillers in the selective DBC domains (see Section 3.2.2) that facilitates the formation of the filler percolation clusters.

As shown in Figure 7a, at higher temperatures close to the ODT point (Δα=0.2), the fillers are diffusely distributed throughout the DBC system. At larger Δα=1.5, the significance of the polymer-filler and filler-filler interactions slightly increases. This effect results in the preferential localization of fillers within the selective *A*-domains (shown in red) and at the interfaces between the domains *A* and *B* (see Figure 7b). This type of filler localization, observed for moderate values of Δα, is due to the comparable significance of the competitive surface and osmotic effects described in Section 2.3. Notably, the case Δα=1.5, shown in Figure 7b, corresponds exactly to the percolation threshold in the filler system for the specified parameter values (see Section 3.2.4). In the case of moderate Δα=8.6, illustrated in Figure 7c, a pronounced localization of fillers within the selective *A*-domains is observed.

The localization of fillers within the selective microphases, observed in the above-described case (c), is consistent with both experimental findings [36] and previous simulations [37,54]. In these studies, sufficiently small fillers are shown to localize within the lamellar domains that have a greater affinity for them. Interestingly, the lamellar structure was found not to be disrupted by the presence of the fillers, which supports the assumptions adopted in our model.

### 3.3. Conductivity of Filled DBC System

The obtained equilibrium localization of conductive fillers in a DBC system, investigated in the preceding Section 3.2.1, Section 3.2.2, Section 3.2.3, Section 3.2.4, has been found to essentially depend on the four main factor, as follows: (i) the difference (contrast) between the affinities of fillers for dissimilar copolymer blocks; (ii) interaction between fillers; (iii) temperature of the composite; (iv) morphology of the DBC system. As the localization of fillers determines the structure of the filler network formed in the composite, the conductivity of this composite essentially depends on the above four factors. This dependence is discussed in the present subsection in detail.

#### 3.3.1. Effect of the Filler Affinity Contrast

**Figure 8:**
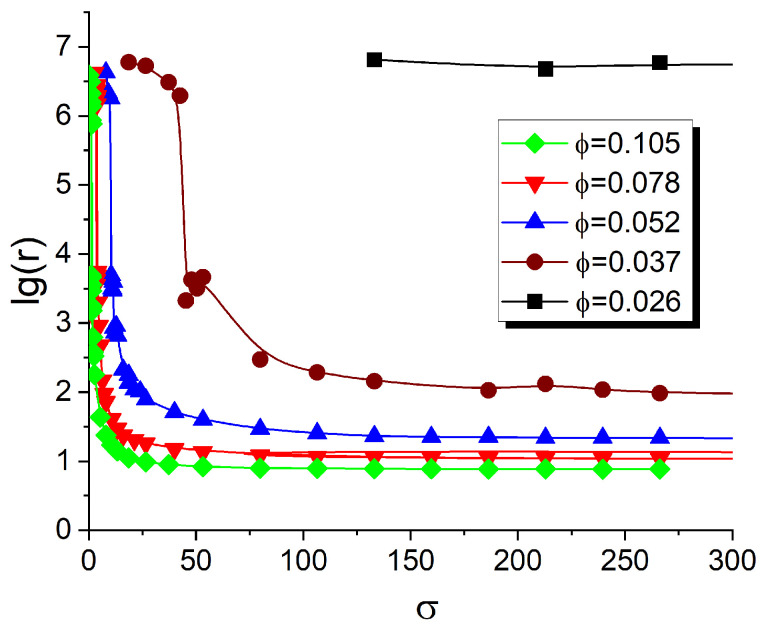
Effect of the difference between the affinities of fillers for dissimilar copolymer blocks quantified by σ on the resistivity of the filled DBC for several selected volume fractions ϕ of fillers.

As shown in Section 3.2.1, the tendency of fillers to localize within the DBC *A*-domains, having larger affinity for these fillers, increases significantly as the value of σ rises from 5 to 133, across all studied filler volume fractions. To systematically study this affinity contrast-induced effect on the conductivity of the composite, we calculated the reduced resistivity *r*, as defined in Section 2.5, as a function of the affinity contrast σ and filler volume fraction ϕ. The results of this calculation are illustrated in Figure 8. Each curve in this figure shows the logarithm of the resistivity (log-resistivity) of the DBC composite as a function of σ for a selected value of ϕ. For each ϕ chosen from the selected set of values (0.026, 0.037, 0.052, 0.078, 0.105), σ is incrementally increased over the range 0<σ<300 through a series of consecutive simulation runs.

According to the simulation results, the composite containing a filler volume fraction of ϕ=0.026 exhibits negligible conductivity 1/r across the entire investigated σ range. The rest of the curves illustrating the cases of larger filler volume fractions (i.e., 0.037, 0.052, 0.078, 0.105) show similar qualitative trends, with variations only in quantitative details. Increasing the affinity contrast σ from zero up to a certain threshold value $\sigma_{tr}\left(\phi \right)$, specific to each of the above filler volume fractions, has no observable effect on the conductivity, so that the composite remains insulating. In Figure 8, the threshold values $\sigma_{tr}$ correspond to the abscissas at which steep drops in log(r) occur. When σ reaches $\sigma_{tr}$, the number of contacts between fillers becomes sufficient to surpass the percolation threshold of the filler network, enabling the flow of electrical current through the composite.

As can be elucidated from Figure 8, the threshold value of σ, delineating the insulating and conducting states of the composite, decreases with increasing filler volume fraction ϕ. This observation leads to the qualitative conclusion that at lower filler volume fractions, the conductor–insulator transition requires a larger difference in the affinities of fillers for the dissimilar copolymer blocks. Further increasing σ beyond the identified conductor–insulator threshold initially results in a rapid decrease in composite resistivity at intermediate σ values, followed by a saturation of resistivity at a limiting value specific to each ϕ. The described behavior of r(σ), as shown in Figure 8, can be explained by the fact that increasing σ enhances the local concentration of fillers within the selective *A*-domains, thereby increasing the number of contacts between fillers. This effect eventually saturates at a characteristic value $\sigma =\sigma_{s}∼150$, beyond which most fillers are already localized in the *A*-microphase. Therefore, any further increase in σ above $\sigma_{s}$ does not significantly affect filler localization or the resulting composite conductivity.

Figure 6a illustrates the relationship between the resistivity of a composite containing a filler volume fraction ϕ=0.078 and the localization of these fillers within the cylindrical morphology of an asymmetric DBC (f=0.45). The localization is primarily governed by the filler affinity contrast for the dissimilar copolymer blocks, which is quantified by the relative adhesion energy per unit area, γ, as defined below Equation (6). A negative (positive) value of γ indicates a stronger affinity of the fillers for the majority (*B*) [minority (*A*)] microphase.

As shown in Figure 6a, for small values of |γ|∼1 mJ/m^2^, the composite remains completely insulating. In this low-affinity contrast regime, fillers are diffusely distributed throughout the DBC matrix, and their local concentration is insufficient to form conductive clusters. When γ, whether negative or positive, is sufficiently large, fillers become predominantly localized within the respective majority or minority microphase, so that the composite becomes conductive. The conductivity increases with increasing |γ|, as a higher absolute affinity enhances the local concentration of fillers in the selective DBC microphases. The composite exhibits higher conductivity for positive γ than for negative γ of the same magnitude. This trend can be observed by comparing points **b**, **c**, and **d** in subplot (a) of Figure 6, which correspond to filler localization patterns shown in the correspondingly marked subplots (see also the discussion in Section 3.2.3). More specifically, the resistivity of the composite with fillers localized in the majority microphase is approximately twice as high as in the case where fillers are localized within the cylindrical domains of the minority *A*-microphase (e.g., point **b** in Figure 6a). This difference is attributed to the larger relative volume of the majority microphase, which leads to a more diffuse distribution of fillers compared to the denser packing observed in the minority phase for the same absolute value of γ.

Interestingly, the resistivity of the filler system with a stronger affinity for the *A*-microphase decreases much more sharply with rising affinity contrast |γ| compared to the case where fillers are localized in the majority *B*-microphase. While the resistivity of the filler network confined to the cylinders of the *A*-microphase reaches its minimum at γ∼20 mJ/m^2^, the corresponding minimum for the *B*-microphase-localized filler system occurs at γ∼−60 mJ/m^2^. This leads to the conclusion that filler localization within the minority *A*-microphase cylinders is more effective for forming a percolating network. These findings further support the conclusion that selective localization in the minority *A*-microphase is more efficient in terms of promoting the formation of a percolating filler network.

#### 3.3.2. Effect of the Interaction Between Fillers

As has been explained in Section 3.2.2 in detail, the attractive (repulsive) interactions between fillers promote (suppress) the selective localization of these fillers in the *A*-microphase of the DBC system. To quantitatively elucidate this effect, we calculated the resistivity of the composite for several selected values of the reduced inter-filler interaction energy βU, while keeping fixed the rest of the parameters (i.e., the affinity contrast of fillers for dissimilar copolymer blocks, DBC segregation and fraction). To isolate the mentioned effects of the composite temperature (segregation), affinity contrast, and filler size, it is instructive to use the affinity parameter that does not depend on Δα and *R*. Such parameter is defined by $\Gamma =\pi b^{2}\gamma /N\Delta w$, $\Delta w\equiv w_{AB}-1/2\left(w_{AA}+w_{BB}\right)$ being the average energy gain from the contact between the dissimilar monomers *A* and *B*. $w_{XY}$ (X,Y=A,B) are the energies of the interaction between monomers *X* and *Y*.

**Figure 9:**
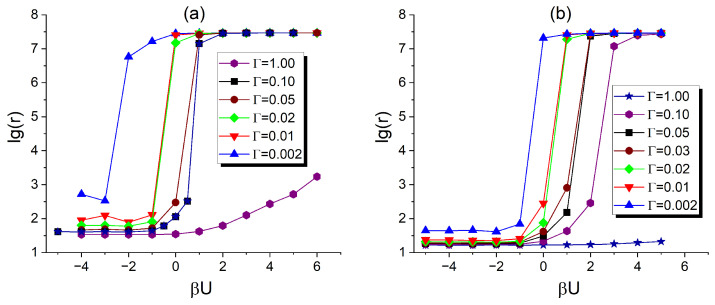
Effect of the interaction between fillers on the resistivity of the filled DBC system for several selected values of the affinity parameter Γ and filler volume fraction ϕ: (**a**) ϕ=0.052. (**b**) ϕ=0.105. See the explanation in the text.

The results of the described calculations for the DBC lamellar microphase are presented in Figure 9. This figure shows the dependence of the composite log-resistivity, log(r), on the reduced inter-filler interaction energy, βU, for two different filler volume fractions: 0.052 and 0.105. Each data set in panel (a) [(b)] of Figure 9 corresponds to the function log(r)(βU) for ϕ=0.052 [ϕ=0.105] and a selected value of the affinity parameter Γ∼γ, as specified in the figure legend.

As shown in Figure 9, sufficiently strong repulsive interactions between fillers (e.g., βU∼5) counteract the effect of selective interactions between the fillers and the copolymer *A*-block. This leads to a decrease in the composite’s conductivity with increasing βU, indicating a more diffuse distribution of fillers compared to that observed at lower βU values. This repulsion-induced dispersion effect is found to be less pronounced at higher affinity contrasts Γ, which tend to dominate the behavior by promoting strong localization of fillers within the *A*-microphase.

Figure 9 also highlights the important role of the filler volume fraction in determining the outcome of the competition between repulsion-driven dispersion and affinity-driven localization. For a higher filler volume fraction (ϕ=0.105), even relatively strong repulsive interactions (U∼5kT) are not sufficient to significantly suppress the composite’s conductivity when the affinity parameter is relatively large (Γ∼1.0). In contrast, for a lower filler fraction (ϕ=0.052), even a relatively weak repulsive interaction (U∼kT) markedly reduces conductivity, reflecting a strong suppression of selective filler localization in the *A*-microphase.

As seen in Figure 9, attractive interactions between fillers significantly reduce the resistivity of the composite across all investigated filler volume fractions. These attractive interactions enhance the crowding of fillers within the selective lamellar *A*-domains, which exhibit a stronger affinity for the fillers. This crowding effect naturally leads to an increase in the composite’s conductivity. At sufficiently high affinity contrast Γ, even relatively weak inter-filler attraction (U∼−kT) is enough to bring the composite’s conductivity to its maximum value for a given filler volume fraction ϕ. This observation is consistent with the results shown in Figure 5d, where pronounced clustering of fillers is evident within the selective *A*-microphase. This clustering results from the interplay of two key factors. First, fillers preferentially localize within the microphase that exhibits higher affinity for them, leading to the formation of dense swarms where the average distance between fillers is much smaller than that expected for a uniform distribution. Second, the reduced separation between fillers within these swarms amplifies the effect of short-range attractive interactions among the fillers, thus promoting the formation of filler clusters. These clusters, observed in Figure 5d, contribute significantly to the reduction in composite resistivity shown in Figure 9.

**Figure 10:**
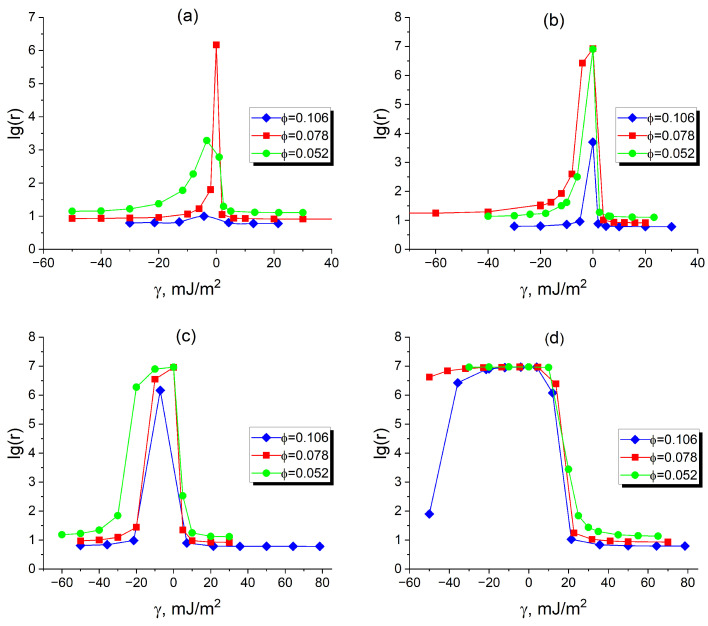
Reduced resistivity of the microphase-separated asymmetric DBC system (f=0.45), which assumes cylindrical morphology, as a function of the filler affinity contrast for dissimilar copolymer blocks, γ, for selected inter-filler interaction energies, *U*: (**a**) βU=−5.0; (**b**) βU=−1.0; (**c**) βU=1.0; (**d**) βU=5.0. Reduced segregation is set to Δα=2.0. The volume fraction of fillers, ϕ, is set equal to the values shown in the legend. See the explanation in the text.

Figure 10 illustrates the effect of inter-filler interactions on the resistivity of a filled asymmetric DBC system, which adopts the same cylindrical morphology as that shown in Figure 6. As in the case of the lamellar morphology discussed previously, the resistivity (conductivity) of the composite strongly depends on the localization of fillers. Recall that in the considered DBC system, the *A*(*B*)-microphase is the minority (majority) microphase. The simulations described in the preceding paragraph have been performed for the selected filler volume fractions (ϕ=0.052, 0.078, 0.105) and the filler-filler interaction energies U=±1kT and ±5kT. According to the simulation results shown in Figure 10, the effect of the interaction between fillers on their localization and conductivity significantly depends on the sign of the affinity contrast γ. For the case of negative γ, when the fillers have larger affinity for the majority *B*-microphase, the sign of the interaction between fillers has a significant effect on the composite conductivity. For the case of relatively large repulsion βU=5.0 between fillers shown in Figure 10d, the observed conductivity is vanishingly small for γ < ∼ 5 mJ/m^2^ at ϕ=0.052 and 0.078. It must be noted that the described effect of the repulsive interaction is not capable to completely suppress conductivity at a larger filler fraction of 0.105 and relatively large affinities γ<−50 mJ/m^2^ of fillers for the majority microphase. Note that the described suppressing effect of the repulsive interactions is less pronounced for smaller βU=1.0 (see Figure 10c). In this case, the non-zero conductivity of the composite is observed in both cases when the affinity of fillers is sufficiently large for either the minority or majority microphase.

The attractive interaction between fillers causes an effect opposite to that of the repulsive interactions described in the preceding paragraph. The corresponding cases are illustrated in Figure 10a,b. As shown in these figures, the attractive interactions increase the conductivity of the composite as they promote the clustering of fillers in the selective DBC microphase. When the affinity contrast γ of the fillers for unlike polymer species is not sufficiently large (γ < ∼ 1 mJ/m^2^), the fillers are distributed in DBC duffusively. This case corresponds to the central peaks of the reduced resistivity in Figure 10a,b in the vicinity of γ∼0 that indicate the insulating state of the composite. Note that this peak is totally absent in the case of larger filler volume fractions 0.078 and 0.105 (0.105) for the case of attractive potential βU=−5.0 (βU=−1.0). This observation indicates that the composite is always conductive in the described cases of relatively large filler volume fractions. As is expected, the attractive interactions have more pronounced effect for larger filler volume fractions. This observation is attributed to the fact that at the larger concentration of fillers they have larger probability to experience the attractive interaction, which increases their clustering.

#### 3.3.3. Effect Caused by Changing the Composite Temperature

**Figure 11:**
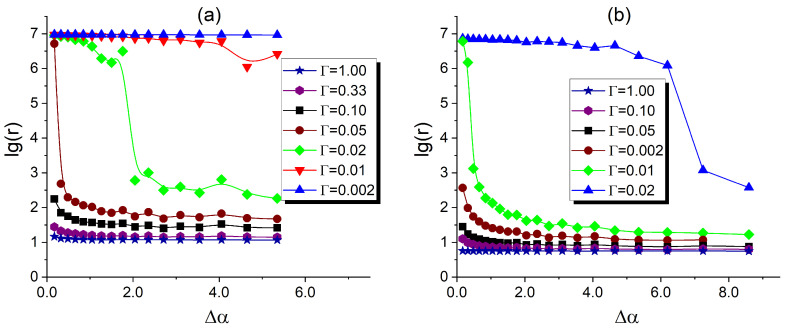
Reduced resistivity of the microphase-separated symmetric (f=0.5) DBC system as a function of the reduced segregation parameter Δα for several selected values of the affinity parameter Γ and filler volume fraction ϕ: (**a**) ϕ=0.052. (**b**) ϕ=0.105. See the explanation in the text.

As shown in Section 2.5, the localization of fillers is strongly influenced by the DBC morphology, which can, in turn, be altered by changing temperature. There is, therefore, an indirect relationship between the temperature of a filled DBC system and its conductivity. Figure 11a,b illustrates the case of the symmetric DBC filled with a volume fraction of 0.052 (0.105) of fillers with a radius of $0.07R_{G}$. These figures show the dependence of the reduced log-resistivity of the composite on the reduced segregation parameter Δα, which quantifies the composite temperature. Scatter plots with point markers in different symbols correspond to different values of the parameter Γ, which quantifies the contrast between the affinities of fillers for dissimilar copolymer blocks. As can be seen from Figure 11, the conductivity of the composite tends to zero at the ODT boundary for all the investigated values of the affinity contrast. Since the fillers are homogeneously distributed in the random DBC morphology formed immediately below the ODT, the effect of the selective localization of fillers promoting non-zero conductance is absent. For sufficiently large values of the affinity contrast Γ and filler volume fraction ϕ, the ODT in the host DBC system was, therefore, found to co-occur with the conductor-insulator transition in the filler system.

At relatively large affinity contrast, Γ∼1, the resistivity reaches its minimum value, which only slightly depends on Δα. This minimum value is found to differ for different volume fractions of fillers, so that, for instance, lg(r)∼0.4 (0.1) for ϕ=0.052 (0.105). This observation is attributed to the fact that a relatively large Γ∼1 provides a sufficient enthalpic gain for the fillers to be almost entirely localized in the selective *A*-microphases. At such a large affinity contrast, the enthalpic effect dominates over the temperature (entropic) effect. With increasing the relative role of the entropic effect associated with increasing temperature (decreasing Δα), the distribution of fillers becomes more diffuse. This effect is particularly evident in the observed increase in resistivity as Δα decreases for all investigated filler volume fractions. A similar trend of increasing resistivity is observed when the affinity contrast Γ decreases, reducing the enthalpic gain from the selective localization of fillers. This trend can be clearly observed by comparing the scatter plots corresponding to smaller values of Γ, shown in Figure 11 for both investigated filler volume fractions.

#### 3.3.4. Effect of the DBC Morphology

**Figure 12:**
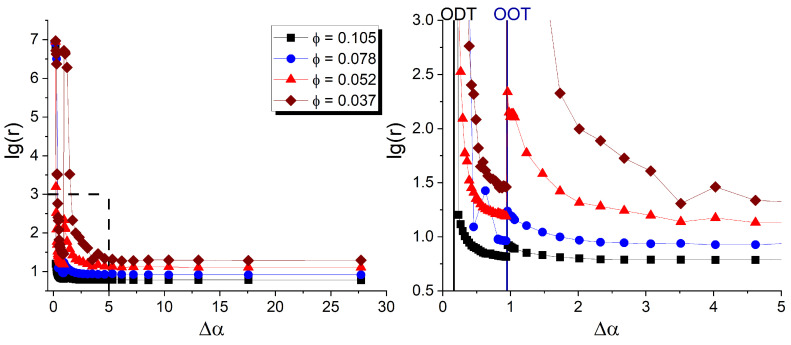
**Left** panel: Reduced resistivity of the microphase-separated asymmetric (f=0.45) DBC system as a function of the reduced segregation parameter Δα for several selected values of the filler volume fractions ϕ. **Right** panel shows a zoom of the selected portion of the **left** panel.

Since the conductivity of the composite was found to be strongly affected by the morphological changes in the DBC lamellae induced by changing temperature, it is instructive to study how a similar effect works for different geometries of the DBC microphases. With this objective, we have calculated the resistivity of the composites under the same conditions, apart from the DBC fraction *f*. Figure 12 shows the resistivity of the composite for the case of asymmetric (f=0.45) DBC, which assumes cylindrical or lamellar morphology. Each point in Figure 12 represents a single simulation set performed for the corresponding values of filler volume fraction ϕ and reduced segregation parameter Δα. Each sequence of simulation sets was performed for a fixed filler volume fraction chosen from the set of values (0.037, 0.052, 0.078, 0.105). γ, the difference in per-unit-area adhesion energies between fillers and dissimilar DBC blocks, and βU, the reduced interaction energy between fillers, are set equal to 5 mJ/m^2^ and −1, respectively, for all the simulation sets shown in Figure 12. The DBC lamellar (cylindrical) morphologies were generated by incrementally increasing Δα in the interval [0,30], while keeping the composition f=0.45 constant.

At relatively large Δα∼20, away from the ODT point, the dependencies of the reduced log-resistivity lg(r) on Δα, depicted in Figure 12, show similar qualitative behavior for all the studied filler volume fractions. Specifically, lg(r) is almost independent of the DBC segregation in the region $\Delta \alpha >\Delta \alpha_{tr}$, where lg(r) reaches its minimum value. Note that the threshold value $\Delta \alpha_{tr}∼5$ strongly depends on the filler volume fraction and the affinity contrast of the filler for dissimilar blocks, quantified by γ. The observed behavior of lg(r) at relatively strong segregation ($\Delta \alpha >\Delta \alpha_{tr}$) can be rationalized by recognizing that, in this region, the DBC forms clear-cut lamellar morphologies separated by narrow interfaces. The microphases with a larger affinity for the fillers form “containers” for these fillers, facilitating their denser concentration. The described localization of fillers is most favorable for the formation of the conductive network, which explains the observed maximal conductance of the composite at $\Delta \alpha >\Delta \alpha_{tr}$.

Upon approaching the order–order transition (OOT) from the lamellar to cylindrical morphology, depicted on the right panel of Figure 12 by the straight line marked “OOT”, the resistivity of the composite increases. This type of behavior occurs in the region $\Delta \alpha_{OOT}<\Delta \alpha <\Delta \alpha_{tr}$. Here, $\Delta \alpha_{OOT}$ corresponds to the OOT point and is evaluated to be 0.96 according to our simulation results for f=0.45. In the described region, the enthalpic gain from the localization of fillers in the selective *A*-microphases is comparable with the osmotic energy gained from the placement of fillers at the interfaces (see Section 2.2 for an explanation). The distribution of fillers in this case, therefore, becomes more diffuse, as the fillers are also spread in relatively wide interfaces formed close to the OOT point. This effect increases the resistivity of the composite, as observed in Figure 12.

The most interesting effect observed in Figure 12 is the abrupt decrease in the resistivity of the composite upon crossing the OOT point, $\Delta \alpha_{OOT}$. Recall that this point delineates the regions corresponding to the lamellar and cylindrical morphologies of DBC. One can therefore conclude that the observed spike in resistivity is associated with the order–order transition (OOT) from the cylindrical to lamellar microphase of DBC. By comparing the composite resistivities below and above the OOT point in its immediate vicinity, one can conclude that the resistivity of the filler network formed in the cylindrical microphase of DBC is several times smaller than that of its counterpart formed in the lamellar microphase. The cylindrical morphology, therefore, is found to promote the composite conductivity. This observation is attributed to the less branched structure of the filler conductive clusters formed in the concise cylinders of the minority microphase. These clusters form shorter conductive paths relative to those observed in the lamellar phase, thus promoting the composite conductivity.

## 4. Conclusions

The present work summarizes and extends our recent achievements in studying the conductivity of a composite composed of a microphase-separated diblock copolymer (DBC) system and conductive fillers. Technically, the reported work relies on a multi-scale approach that combines the phase-field model of the DBC with Metropolis Monte Carlo (MC) simulations of the filler system immersed in this DBC system. The developed phase-field model is described in detail in Section 2.2, along with the method for solving the derived phase-field equations. On the microscale, the model accounts for the microscopic interactions between fillers and polymers, as well as those between the fillers themselves. An important feature of these interactions, which is quantitatively analyzed in Section 2.3, is their dependence on the location of the fillers in the DBC. Furthermore, the microscopic interaction between fillers and polymers has been incorporated into the solution of the phase-field equations to determine the immersion energy of a filler depending on its position within the DBC system. The calculated immersion energy enables the sampling of possible filler localizations in the DBC, as the position-dependent immersion energy varies for different filler localizations. The details of this sampling and the performed MC simulations are described in Section 2.4. Based on the predicted selective localization of fillers, a random network model was constructed to mimic the conductive filler network formed in the DBC. This random network was then used to calculate the resistivity of the composite under different conditions, as described in Section 2.5.

The performed analysis aims to identify the main factors influencing the conductivity of the described composites. These factors are found to be as follows: (i) affinities of fillers for copolymer blocks; (ii) degree of the segregation of a host DBC system, driven by the composite temperature; (iii) geometry of the microphases formed in the microphase-separated DBC; (iv) interactions between fillers. The role of these factors in tailoring the composite’s conductivity is quantitatively analyzed and described in Section 3.2.1, Section 3.2.2, Section 3.2.3, Section 3.2.4. Below, we summarize the qualitative trends deduced from these findings.

The key factor affecting both the localization of fillers in the DBC system and the conductivity of this system is the immersion energy of the fillers in the DBC. This energy has two contributions with distinct physical natures. The first contribution comes from the osmotic (excluded volume) interactions between the fillers and polymers. This contribution favors the placement of fillers at the interfaces between the DBC microphases, where they screen unfavorable interactions between the dissimilar copolymer blocks. The second contribution is enthalpic in origin, caused by the difference in the affinities of fillers for the dissimilar copolymer blocks. This enthalpic contribution dominates over the osmotic contribution when the described difference is sufficiently large. Changing the relative roles of the described osmotic and enthalpic contributions (e.g., through the surface treatment of fillers) can be used to achieve the desirable localization of fillers in DBC, such as interfacial or central-microphase localization. This theoretical conclusion, derived from our simulations, explains the experimental observations of the localization of gold fillers in DBC PS-b-P2VP, as demonstrated in Figure 4.

The second important factor affecting the composite conductivity is the degree of segregation of the DBC, which can be driven by external stimuli such as changes in the composite temperature. In particular, sufficiently strong segregation of the symmetric DBC results in the formation of distinct lamellae separated by narrow interfaces. These morphological structures, composed of DBC blocks that have larger affinities for fillers, serve as “containers” for these fillers (see Figure 7c). This strongly segregated DBC morphology, therefore, facilitates the formation of conductive filler networks even at relatively small filler volume fractions (∼3%). At temperatures slightly above the order-disorder transition (ODT) point, the DBC becomes weakly segregated, featuring wider lamellae separated by broader interfaces. In this regime, fillers become localized in a larger volume, thus creating a less dense conductive network in the selective lamellae (see Figure 7b). This effect results in a reduction of the composite conductivity at higher temperatures (see Figure 11). An additional factor enhanced by the increasing temperature is the increase in the translational entropy of the fillers, which promotes their more diffuse distribution throughout the DBC system. Our simulations show that at the ODT point, where the DBC undergoes the order-disorder transition, the conductor-insulator transition in the filler network occurs (see Figure 11). This effect is attributed to the fact that, near the ODT, the fillers are no longer contained within the selective DBC microphases. The concentration of fillers in the vicinity of ODT is, therefore, no longer sufficient to provide for the formation of the conductive network.

The third important factor affecting the conductivity of the filler network formed in DBC is the geometry of the DBC microphases. To analyze the significance of this factor, we compared the conductivity of symmetric and asymmetric DBCs containing the same amount of fillers with the same affinities for the copolymer blocks. This analysis showed that cylindrical microphases provide better conditions, compared to lamellar morphology, for the formation of the conductive filler network. This effect is especially pronounced when the fillers have larger affinities for the minority microphase forming the cylinders (see Figure 6). In this case, the conductivity of the filler network localized in the cylindrical microphases reaches its maximum at a relatively low affinity contrast (∼20 mJ/m^2^) of these fillers for the dissimilar copolymer blocks. For the case when the fillers have a larger affinity for the majority microphase, a similar effect is achieved at a much larger affinity contrast (∼60 mJ/m^2^). The most important difference between the conductivity dependencies of the symmetric- and asymmetric DBC-based composites on their temperature is caused by the presence of an additional order-order transition in the asymmetric DBC. Specifically, the conductivity of the composite based on the microphase-separated asymmetric DBC experiences a jump as a function of DBC segregation (temperature). The observed spike in conductivity (see Figure 12) is found to be caused by the restructuring of the filler conductive network associated with the transition between the lamellar and cylindrical morphologies. The observed higher conductivity of the filled asymmetric DBC system is caused by a more efficient structural organization of the filler clusters in the cylindrical DBC microphases, which facilitates conducting electrical current.

The fourth important factor involved in tailoring the electrical response of a filled DBC system is the interaction between fillers. The general trends imposed by this interaction, as deduced from our simulation results, can be summarized as follows: The repulsive (attractive) interaction between fillers suppresses (promotes) the conductivity of the composite, as this interaction works to deplete (enhance) the local density of fillers in the selective DBC microphases. It is important to note that the described interaction factor is interrelated with the other influencing factors described in the preceding three paragraphs. In particular, the relative significance of the attractive interaction between fillers essentially depends on the affinity of these fillers for dissimilar copolymer blocks. According to our results (see Figure 5d), when the affinity of fillers for one copolymer block is significantly larger, the attractive interactions lead to pronounced clustering of these fillers, which promotes the conductivity of the composite. Moreover, the effect of the interactions between fillers on composite conductivity depends on the geometry of the microphases adopted by DBC. Specifically, this effect has different significance depending on whether the minority or majority DBC microphase has a greater affinity for fillers. When the affinity of the fillers is greater for the majority microphase, a sufficiently strong repulsive interaction between fillers suppresses the conductivity of the composite (see Figure 5d). Strong repulsive interactions between fillers dilute the filler concentration and, therefore, prevent the formation of conductive clusters. Interestingly, repulsive interactions of the same strength are not sufficient to suppress the conductivity of the filler network formed inside the concise cylinders of the minority microphase. In contrast, attractive interactions enhance the conductivity of the composite when a sufficient fraction of fillers is contained in either the minority or majority microphase (see Figure 5a,b). This effect is especially strong for a moderate volume fraction of fillers, ϕ∼0.1, localized in the majority microphase. Generally, the effect of interactions between fillers is less pronounced when the fillers are localized in the cylinders of the minority microphase.

In summary, the present work identifies the main factors involved in promoting or suppressing the conductivity of a composite composed of a host DBC system and conductive filler particles. The majority of these factors can be enhanced or reduced, depending on practical needs, by performing relatively straightforward manipulations on the fillers or host polymer systems. For instance, a significant effect on the composite’s conductivity can be achieved by temperature- or pressure-induced reversible alterations to the DBC morphology. Surface modification of the filler surfaces, performed through surface treatment [21] or grafting ligands [40], can also be effectively used to change the localization of fillers and, through this, the electrical response of the composite. The multi-scale approach developed in the current work provides a theoretical basis for designing DBC-based nanocomposites with controlled electrical responses for applications, e.g., in flexible electronics [62,63] and soft sensors [19,64,65,66,67].

## Data Availability

Data are contained within the article.

## Abbreviations

The following abbreviations are used in this manuscript:

DBCs	diblock copolymers
MC	Monte Carlo
OOT	odrer–order transition
r.(l.)h.s.	right (left)-hand side
